# Gradients of Orientation, Composition, and Hydration of Proteins for Efficient Light Collection by the Cornea of the Horseshoe Crab

**DOI:** 10.1002/advs.202203371

**Published:** 2022-10-17

**Authors:** Oliver Spaeker, Gavin J. Taylor, Bodo D. Wilts, Tomáš Slabý, Mohamed Ashraf Khalil Abdel‐Rahman, Ernesto Scoppola, Clemens N. Z. Schmitt, Michael Sztucki, Jiliang Liu, Luca Bertinetti, Wolfgang Wagermaier, Gerhard Scholtz, Peter Fratzl, Yael Politi

**Affiliations:** ^1^ Department of Biomaterials Max Planck Institute of Colloids and Interfaces 14476 Potsdam Germany; ^2^ Institute for Globally Distributed Open Research and Education (IGDORE) Ribeirão Preto 14091‐310 Brazil; ^3^ Chemistry and Physics of Materials University of Salzburg Jakob‐Haringer‐Str. 2a Salzburg 5020 Austria; ^4^ Adolphe Merkle Institute University of Fribourg Chemin des Verdiers 4 Fribourg 1700 Switzerland; ^5^ TELIGHT Libušina třída 21 Brno 623 00 Czech Republic; ^6^ European Synchrotron Radiation Facility (ESRF) 71 avenue des Martyrs, CS 40220 Grenoble Cedex 9 38043 France; ^7^ B CUBE – Center for Molecular Bioengineering Technische Universität Dresden 01307 Dresden Germany; ^8^ Humboldt‐University Berlin Institute of Biology Philippstraße 13 10115 Berlin Germany

**Keywords:** biomaterials, chitin‐based materials, optical materials, optical modeling, protein composition, structure–function relationships, vision

## Abstract

The lateral eyes of the horseshoe crab, *Limulus polyphemus*, are the largest compound eyes within recent Arthropoda. The cornea of these eyes contains hundreds of inward projecting elongated cuticular cones and concentrate light onto proximal photoreceptor cells. Although this visual system has been extensively studied before, the precise mechanism allowing vision has remained controversial. Correlating high‐resolution quantitative refractive index (RI) mapping and structural analysis, it is demonstrated how gradients of RI in the cornea stem from structural and compositional gradients in the cornea. In particular, these RI variations result from the chitin‐protein fibers architecture, heterogeneity in protein composition, and bromine doping, as well as spatial variation in water content resulting from matrix cross‐linking on the one hand and cuticle porosity on the other hand. Combining the realistic cornea structure and measured RI gradients with full‐wave optical modeling and ray tracing, it is revealed that the light collection mechanism switches from refraction‐based graded index (GRIN) optics at normal light incidence to combined GRIN and total internal reflection mechanism at high incident angles. The optical properties of the cornea are governed by different mechanisms at different hierarchical levels, demonstrating the remarkable versatility of arthropod cuticle.

## Graphical abstract

Vision is well studied in the Atlantic horseshoe crab, *Limulus polyphemus*, whose lateral compound eyes are the largest within recent Arthropoda and although they are well understood from a neurophysiological perspective, it is unclear what determines its optical properties. Here, it is shown that these are determined by the organization of chitin and proteins, including variations in protein composition and hydration.

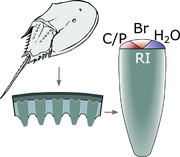

## 1 Introduction

Arthropod vision has fascinated researchers for more than a century for its diverse optical mechanisms and recently for potential bio‐inspired design of microlens arrays.^[^ ^1^, ^2^ ^]^ Their compound eyes form an array of individual light collecting and sensing units, the ommatidia, typically consisting of a cornea, a conical lens or light guiding device, and a receptor unit called rhabdom.^[^ ^3^ ^]^ Light entering the ommatidium is guided toward the rhabdom by refraction, reflection, or a combination thereof.^[^ ^4^ ^]^ An image is created by the integration of stimuli from each receptor unit, which receives the light from optically isolated ommatidia in apposition eye systems, or multiple neighboring ommatidia in superposition systems.^[^ ^3^ ^]^

**Figure 1:**
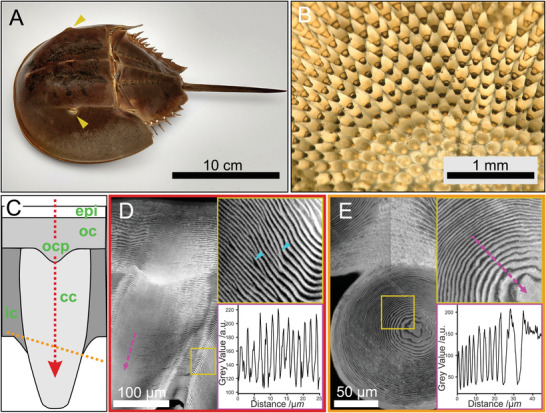
Structure of *Limulus polyphemus* cornea. A) Image of *L. polyphemus* showing the position of the lateral compound eyes (yellow arrowheads). B) Transmission light micrograph taken from the proximal side of a cornea after removal of cells and pigments. Note the dark and bright appearance of the cone depending on the viewing angle. C) Schematic depiction of corneal cones (cc) illustrating the different regions discussed in the text, intercone (ic), epicuticle (epi), outer‐cornea (oc), and outer‐cornea protrusion (ocp). The yellow and red dotted lines indicate the section orientation in (D and E). The arrow on the red line indicates the light propagation direction. D) A longitudinal section (red dotted line in C) stained with DY96 and imaged with confocal laser scanning microscopy (CLSM) readily reveals the helicoidal arrangement of chitin in the *L. polyphemus* cornea, as can be deduced from the sinusoidal alternation of light and dark bands. Upper inset: magnification of the cone‐intercone border, yellow square, showing defects in lamella organization (blue arrowheads). Bottom inset: variation in gray level along the magenta dotted arrow. A section of a full cone shown in Figure S1B (Supporting Information). E) CLSM of DY96‐stained cross‐sections (orange dotted line in C). Upper inset: magnification close to the cone center, marked in yellow square in E. Bottom inset: variation in gray level along the magenta dotted arrow. The bright and dark bands broaden toward the center due to lamellae inclination. The brightness difference around the midline is an image‐stitching artefact.

Vision is especially well studied in the Atlantic horseshoe crab, *Limulus polyphemus* (*L*. 1758, *Xiphosura*).^[^ ^5^ ^]^ Horseshoe crabs have six rudimentary eyes, two ocelli, and a pair of lateral compound eyes (**Figure** **1**A).^[^ ^6^ ^]^ While the latter are very well understood from a neurophysiological perspective, a debate remains as to their light focusing mechanisms.^[^ ^7^, ^8^ ^]^ Exner proposed a radial nearly parabolic refractive index (RI) gradient in the cones, later confirmed and quantified by Land, that leads to a refraction‐based focusing mechanisms, termed cylinder‐lens by Exner and described today as graded Index (GRIN) optics.^[^ ^1^, ^8^ ^]^ Conversely, Levi‐Setti et al. emphasized the cone shape and ascribed the focusing power of the lens to total internal reflection (TIR) at the interface between cornea and surrounding tissue, suggesting it as an example of an optimal light collector.^[^ ^9^ ^]^ Although the shape of the cones and the general radial RI gradient in the lenses are well described, to date little is known about the structure–property–function relationships in the material that forms the lens. Here, we determine how the optical properties of the lens stem from its multiscale architecture and local composition. To that end, we have mapped the RI along and across the cones and correlated the results with spatially resolved structural and compositional analyses of the material. Using different optical modeling approaches, we demonstrate that both the observed GRIN profile and lens shape have roles in determining the overall optical behavior.

## 2 Results and Discussion

The *L. polyphemus* lateral eye consists of an array of lenses forming a cornea and inwards projecting cones (Figure 1B,C and Figure S1, Supporting Information). In contrast to the situation in most insects and crustaceans and presumably representing the ancestral condition for arthropod eyes, the cones are formed by the cuticle, the same material that builds the animal's exoskeleton.^[^ ^10^ ^]^ Arthropod cuticle is made of a chitin‐protein fiber composite that shows structural and compositional versatility which allows its multifunctional roles.^[^ ^11^ ^]^ In *L. polyphemus*, the corneal cones are hierarchically structured: the chitin‐protein fibers are organized helicoidally leading to a lamellated appearance, where the lamellae (i.e., fiber‐sheet layer consisting of half helicoidal pitch) organize in a curved nested arrangement that follows the cone geometry (Figure 1D,E). Image stacks acquired by confocal laser scanning microscopy (CLSM) of Direct Yellow 96 (DY96)‐stained sections of the cornea reveal a regular 3.52 ± 0.49 µm helicoidal pitch across and along the cone, with radially increasing lamella‐inclination from the center to the edge of the cone (Figure 1E). Here, the cones are embedded in a cuticular region, hereafter termed intercone, showing an increased helicoidal pitch of 5.96 ± 1.04 µm. The mismatch in helicoidal pitch dimensions leads to multiple defects, especially concentrated at the interface region between cones and intercone (Figure 1D inset arrowheads). At the distal side, the cones are connected to the outer‐cornea through a structure that we termed the outer‐cornea protrusion, and a thick epicuticle constitutes the interface with the environment (Figure 1C,D). The arthropod cuticle is typically perforated by multiple pore‐canals that serve for transport across the cuticle. In *L. polyphemus* compound eyes, pore‐canals are only present in the intercone but not within the cones or other regions of the cornea (Figure 1D,E and Figure S1C, Supporting Information).

**Figure 2:**
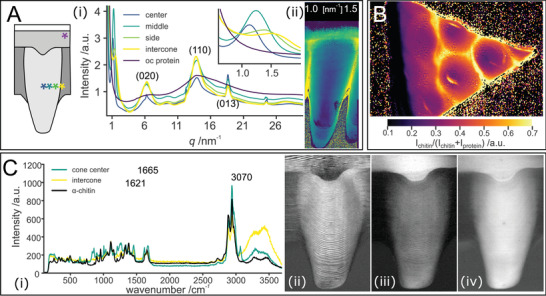
Molecular structure and compositional variations along the cornea. A) Scanning X‐ray diffraction (XRD)/small‐angle scattering (SAXS) mapping. i) Representative XRD profiles obtained by averaging diffraction patterns at different regions of the *Limulus polyphemus* cornea measured in dry state. The inset shows a magnification of the chitin correlation peak around 1.1 nm^−1^. The positions within the cornea are indicated by color‐coded asterisks in the cornea scheme on the left. ii) Mapping the *q*‐position of the correlation peak across a dried longitudinal section. B) Fourier transform infrared spectroscopy (FTIR) map of a dry oblique section of the cornea containing the outer‐cornea. The map depicts the ratio of the chitin and protein signals as determined from the intensity ratio in the regions: 1700 to 1600 cm^−1^ and 1180 to 1000 cm^−1^, Figure S3C (Supporting Information). C) Micro‐Raman spectroscopy; i) averaged spectra of the cone and intercone regions in comparison to pure *α*‐chitin. The center of the peaks used for imaging in (ii–iv) are marked with dotted lines. ii) Mapping the integrated intensity, the chitin amide I peak between 1640 and 1750 cm^−1^ (iii) mapping of the integrated intensity of the beta‐sheet peaks between 1576 and 1635 cm^−1^, and iv) mapping the integrated intensity peak of the CH band of aromatic and aliphatic side chains between 3035 and 3095 cm^−1^.

We investigated the hierarchical structure of the corneal cones at the µm‐ and nm‐scale by scanning X‐ray diffraction (XRD) using cornea thin sections in the hydrated and dry state (**Figure** **2**). The acquired XRD profiles show the typical reflections of *α*‐chitin with the (020) reflection at scattering vector (*q*) between 6.4 to 6.6 nm^−1^, the (110) reflection at *q* = 13.6 nm^−1^ and the (013) at *q* = 18.5 nm^−1^ (Figure 2A).^[^ ^12^, ^13^, ^14^ ^]^ Additionally, in the small‐angle scattering (SAXS) region, a correlation peak resulting from chitin‐protein fiber packing^[^ ^15^ ^]^, is observed at *q* = 1.1 nm^−1^. Scanning the sample using an X‐ray beam with 300 nm cross‐section reveals heterogeneity in the cornea's material composition and structure. Figure 2Ai shows XRD profiles of various regions within the cornea. However, at the outer‐cornea surface, chitin reflections are absent and instead the diffraction profile shows two broad peaks at *q* = 6 and 14 nm^−1^ (Figure 2Ai, violet curve). The occurrence of these peaks is indicative of cross‐beta structures in proteins.^[^ ^16^, ^17^ ^]^ Cross‐beta protein organization is common in arthropod cuticles, however it is often difficult to discriminate the protein reflections from those of chitin as they typically coincide and since chitin‐poor or protein‐rich layers are usually thin with respect to the probed volume.^[^ ^18^, ^19^ ^]^ In *L. polyphemus* an especially thick epicuticle (the part of the cuticle that is devoid of chitin) allows direct insight into the dominant protein structure in the cuticle. The variation in the relative abundance of chitin and protein as suggested by the variation in fiber packing (Figure 2Aii), is also reflected in Fourier transform infrared spectroscopy (FTIR) measurements, which demonstrate in addition the absence of chitin in the outer layers of the cornea (Figure 2B, Figure S3C, Supporting Information).

Using XRD, we identified that the protein matrix contains a large fraction of beta sheets, organized in a cross‐beta structure. However, due to the overlap between chitin and protein reflections, it is difficult to determine their respective arrangement. This difficulty is overcome using micro‐Raman spectroscopy. Figure 2Ci shows the Raman spectra of pure *α*‐chitin, and the averaged spectrum of the cone center and intercone regions. We have chosen to map the intensity of three scattering peaks, one chitin specific peak (1655 cm^−1^), and two related to the proteins (and absent in pure chitin (1621 and 3070 cm^−1^)) (Figure 2C). Due to the polarized nature of the Raman laser, the intensity of peaks originating from highly oriented bonds varies with the respective orientation of the bond and the laser polarization direction. Therefore, the intensity of the chitin amide I peak (1665 cm^−1^) oscillates with the fiber orientation revealing the lamellate structure of the cone (Figure 2Cii).^[^ ^20^ ^]^ The same effect is visible when plotting the beta‐strand peak at 1621 cm^−1^ (Figure 2Ciii), demonstrating the backbone of the proteins is organized in a similar way.^[^ ^20^ ^]^ Mapping the contribution of aromatic side chains (peak at ≈3070 cm^−1^), exposes an overall gradient in intensity from the center to the edge of the cones, but not the lamellate structure (Figure 2Civ).^[^ ^21^ ^]^ This gradient stems from the variation in the overall protein content but may also indicate that the type of specific proteins changes from center to edge of the cone.

**Figure 3:**
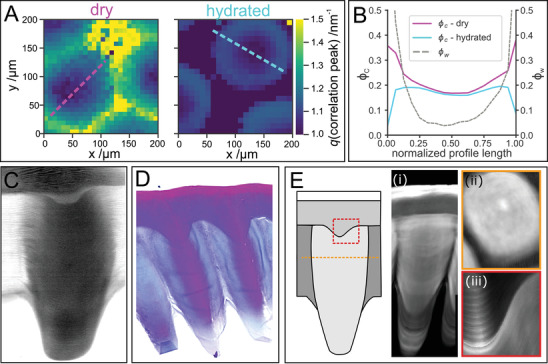
Hydration and sclerotization across the cornea. A) The effect of hydration on the *q*‐position of the fiber correlation peak in cornea cross‐sections. The central values are similar in both samples, whereas toward the edges of the cones they shift to lower *q* in hydrated (right) relative to dry (left) samples, indicating that swelling due to hydration is limited to this region. Dotted lines indicate the positions of the profiles shown in B. B) Chitin and water volume fractions calculated from the correlation peak position extracted from maps in A (Equation 1). The dimensions of the cones are normalized to accommodate for the different dimensions between dry and hydrated state. C) Micro‐Raman spectroscopy hydration map obtained by plotting the integrated intensity of the OH bands between 3111 and 3689 cm^−1^. D) Mallory trichrome staining of a longitudinal cornea section. E) Br X‐ray fluorescence (XRF) maps of the cornea as indicated in the schematic on the left. i) High‐resolution XRF maps of Br K*α* of a cone longitudinal section. The section is cut off‐axis therefore it does not contain the outer‐cornea protrusion and ii) a cross‐section showing bright cones in darker intercone matrix. The section is cut along the orange dotted line on the scheme (iii) a longitudinal section at the outer‐cornea protrusion region marked on the scheme with a red frame.

We have used the position of the fiber correlation peak in the SAXS region to estimate the chitin and protein volume fraction across the cones. In air‐dried sections, the *q‐*position of this peak changes along the radial direction of the cones from 1.1 to 1.42 nm^−1^, and increases to 1.5 nm^−1^ in the intercone region **Figure** **3**A, corresponding to a change in fiber‐to‐fiber distance from around 5.7 to 4.6 nm and 4.2 nm, respectively. The chitin volume fraction (*φ*_c,dry_) across the sample can be estimated assuming constant chitin crystallites diameter and a hexagonal packing, using

(1) $$\varphi_{c,\mathrm{RH}}=\frac{\pi }{2\sqrt{3}}{\left(\frac{x}{d_{\mathrm{RH}}}\right)}^{2},$$

where *x* is the chitin crystal diameter (2.84 nm, see SI) and *d*_RH_ is the fiber‐to‐fiber distance for either dry or hydrated samples retrieved from the position of the SAXS peak.^[^ ^22^ ^]^ The chitin volume fraction in dry samples changes from approximately 20% in the cone center to 37% at the cone edge (Figure 3B), where protein volume fraction, *φ*_p,dry_, is assumed as 1 — *φ*_c,dry_. In its natural environment the *L. polyphemus* cornea is fully hydrated. Hydrated cornea sections show an interesting swelling behavior; while the correlation peak shifts to lower *q* values at the edges of the cone in hydrated compared to dry cornea sections, indicating an increased distance between chitin fibrils, the *q* values measured in the center remain unchanged (Figure 3B). The water volume fraction *φ*_w_ was calculated using *φ*_c,dry_, *φ*_p,dry_ and the chitin volume fraction in hydrated cornea cross‐sections, according to the equation

(2) $$\begin{array}{c} \varphi_{w}=1-\varphi_{c,\mathrm{hydrated}}\left(1+\frac{\varphi_{p,\mathrm{dry}}}{\varphi_{c,\mathrm{dry}}}\right) \end{array}$$

The water volume fraction shows a roughly parabolic profile with its minimum close to the cone center (Figure 3B). This hydration gradient can also be visualized using micro‐Raman spectroscopy by mapping the intensity of the OH bands between 3111 to 3689 cm^−1^ (Figure 3C).^[^ ^23^ ^]^

Cuticle hydration is tightly correlated with its sclerotization level, i.e., the incorporation of catechol derivatives that cross‐link the matrix and render it hydrophobic.^[^ ^24^, ^25^, ^26^ ^]^ To visualize the sclerotization level within the cornea, we used Mallory staining. This histological triple stain is sensitive to sclerotization and is typically used to differentiate between cuticle layers: unsclerotized cuticle (e.g., endocuticle) is stained blue, whereas sclerotization, as found in mesocuticle and muscle attachment sites, leads to intense magenta coloration.^[^ ^27^ ^]^ Cones stained with Mallory display strong magenta coloration in their core and a blue‐stained circumference indicating a cross‐linked core and poorly sclerotized shell (Figure 3D), in striking agreement with the hydration results from Raman and XRD.

Concurrently with XRD, we also recorded the emitted X‐ray fluorescence (XRF) (Figure 3E). We detected an elevated Br signal in the epicuticle, as previously observed in insects.^[^ ^18^ ^]^ Br was also present in the cones but not in the outer‐cornea or the intercone region (Figure 3F and Figure S2Bii, Supporting Information). In addition, a thin cuticular layer enriched with Zn surrounded the surface of the cones in the region where they extend out of the intercone layer (Figure S2Ci, Supporting Information). Interestingly, the Br distribution does not closely follow the sclerotization pattern determined by Mallory staining, suggesting that in *L. polyphemus* halogen incorporation is not directly coupled to the sclerotization process as often suggested for insects.^[^ ^25^ ^]^

**Figure 4:**
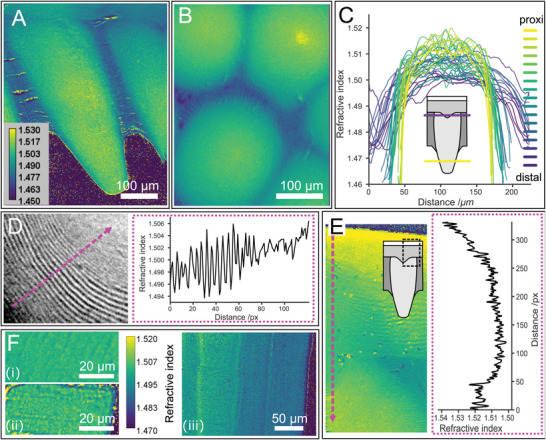
Refractive index (RI) mapping of the cornea. RI maps are calculated from phase maps of a A) longitudinal and B) cross‐section (Same LUT as in A). C) RI profiles of cross‐sections extracted from the base of the cone to approximately 50 µm from the cone tip. Note that the exact height in the cone cannot be determined. The profiles shown were taken from multiple cones in close vicinity to each other within single cornea. D) Structural RI variation observed in high magnification RI map of a cross‐section. The dotted magenta arrow shows the line from which the profile (magenta frame) is taken. E) High magnification RI map of a cornea longitudinal section around the outer‐cornea protrusion (The exact area is depicted in the scheme). Note the correlation with the Br content in different cornea regions shown in Figure 2B. F) RI maps of longitudinal (i) and cross (ii) sections of bleached tendons (almost pure chitin) showing similar RI value. iii) A longitudinal section of native tendon (with native proteins) demonstrating the effect of proteins on RI.

To determine the contributions of the observed structural and compositional heterogeneities to the optical properties of the eyes, we performed spatial mapping of the RI (at 650 nm) in longitudinal and cross‐sections of the cornea using quantitative phase imaging (QPI).^[^ ^28^ ^]^ This method allows quantitative determination of the phase shift of light transmitted through the sample. Using QPI acquired in the presence of two media with different RI (phase decoupling, *n*_m1_ = 1.425, *n*_m2_ = 1.457), a spatially resolved RI map can be calculated with µm‐resolution (**Figure** **4**).^[^ ^29^ ^]^ As determined by Land, a roughly parabolic radial RI gradient across the cone is observed, with a maximum (*n* = 1.52) decreasing to *n* = 1.47 at the edge of the cone (Figure 4A—C), while the RI in the intercone region drops to *n* = 1.44, due to the presence of medium‐filled pore‐canals (see note in SI).^[^ ^8^ ^]^ Furthermore, we observe a longitudinal gradient with increasing RI toward the cone tip (Figure 4C).

Interestingly, the maximum RI determined from QPI analysis was systematically increased in cross‐sections relative to longitudinal sections (Figure S3A, Supporting Information), attesting to a structural effect caused by the helicoidal arrangement of birefringent fibers and their changing inclination across the cone. Indeed, oscillating RI values (with Δ*n* = 0.01) are observed in RI maps obtained at high magnification that coincide with the lamellar texture of the cones (Figure 4D).

Chitin is birefringent due to its orthorhombic crystal structure. Quantitative measurements and modeling of the chitin birefringence have yielded Δ*n* = 0.0024 (at 589 nm).^[^ ^30^ ^]^ Indeed, the measured RI of oriented chitin fibers along and across their fiber axis, using deproteinized *L. polyphemus* tendons, show similar RI (*n* = 1.503 ± 0.005, at 650 nm), within the instrumental resolution, in both directions (Figure 4F). In comparison, intact tendons in which the chitin crystals are decorated by ordered proteins, reveal an RI inhomogeneity that exceed the observed birefringence in the cornea (Figure 4Fiii). These measurements demonstrate that the protein content and composition can drastically alter the RI of the material. The birefringence of ordered fibrous proteins with predominant beta‐sheet structures such as silks lies in the range of Δ*n* = 0.02–0.04, more than one order of magnitude higher than that of chitin.^[^ ^31^, ^32^ ^]^ We thus attribute the structural effect of fiber orientation on the RI to ordered proteins rather than to chitin itself.

The gradient in RI cannot be explained by the structural effect alone. The higher volume fraction of proteins in the center of the cones as determined by XRD and FTIR lead to an increase in RI, while the increased water content at their periphery lowers the RI. Additionally, a sharp increase in RI (Δ*n* = 0.01) is observed in RI maps taken at the outer‐cornea protrusion that correlate with the steep increase in Br K*α* XRF signal (Figure 4E), and the maximum determined RI (*n* = 1.54) correlates with the highest level of Br at the epicuticle. Halogens in general and Br specifically have been used as dopant to increase the RI of organic polymers.^[^ ^33^ ^]^ This suggests that the incorporation of halogens into proteins, which is not uncommon in cuticular materials and has been reported previously for *L. polyphemus* cuticle, may serve here to locally increase the material's electron density and thus its RI.^[^ ^3^ ^]^

**Figure 5:**
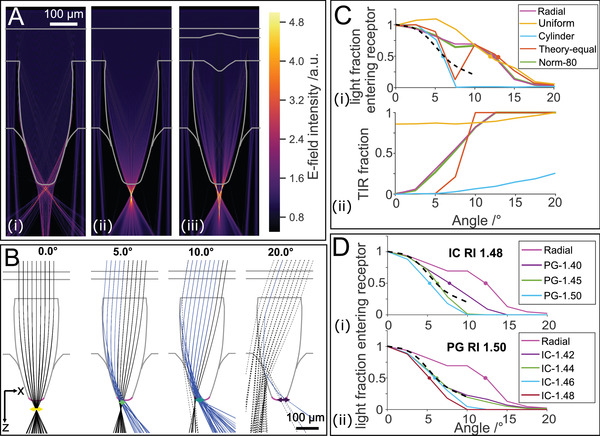
Finite‐difference time‐domain (FDTD) and 3D ray‐tracing simulations. A) FDTD simulations with 650 nm light: i) cone model with uniform refractive index (RI) of 1.52, inner medium RI of 1.34, and intercone RI of 1.4, ii) cone with the observed radial RI profile, and iii) cone model including contributions from all subregions using the RI profile in (ii). B) Ray tracing results projected onto *xz*‐plane for different incident angles with the observed RI profile and intercone value of 1.44 (pink plot in C and D). Rays that undergo total internal reflection (TIR) are plotted in blue, colored horizontal arrows represent the circle of least confusion (COLC). Rays that exit the cone before its tip (pink line) are dashed. C) Acceptance functions based on dark adapted receptor dimensions (inner medium RI: *n* = 1.34, intercone RI: *n* = 1.40) (i). The dashed line represents the physiological results measured by Barlow et al. 1980.^[^ ^39^ ^]^ Full circles indicate the 50% value on each curve, which represent the half‐width at half‐maximum acceptance angle. ii) Fraction of rays that undergo TIR before exiting the cone tip. D) Acceptance functions based on dark adapted receptor dimensions with a fixed RI value for either the i) intercone (IC) or ii) inner medium (PG).

We used full wave optical modeling and ray tracing simulations to systematically determine the role of the RI profiles and cone shape in guiding incident light into the receptor cells.^[^ ^36^, ^37^, ^38^ ^]^ Finite‐difference time‐domain (FDTD) simulations using a cone shape extracted from µCT data and a uniform cone RI (*n* = 1.52), revealed that TIR at the cone tip is sufficient to guide on‐axis light to an aberrated focal point at the cone tip (**Figure** **5**Ai). Introducing the observed radial RI gradient drastically improves the focusing of on‐axis light and places the focal point 30 µm away from the cone tip, to a point that coincides with the distal end of the receptor (Figure 5Aii).^[^ ^6^ ^]^ Implementing other anatomical details with RI variation (outer‐cornea, epicuticle, and the outer‐cornea protrusion) lead to negligible variation in the optical behavior (Figure 5Aiii).

Ray‐tracing simulations corresponded with FDTD results at normal incidence (Figure 5B), varying the incident angle in ray‐tracing simulations showed considerable variation in off‐axis light focusing performance between different models. In particular, a simulated lens cylinder has a much narrower acceptance angle (Figure 5Ci, half‐width at half‐maximum – 5.9°) compared to models using a cone shape, which have acceptance angles around 13°. Although the former is a close match to previous physiological measurements (6.5°), the cylinders acceptance function has a nonphysiological abrupt cut‐off at higher angles of incidence.^[^ ^39^ ^]^ Imposing a cone shape on‐top of the RI profile of the idealized lens cylinder broadened its acceptance function considerably although, surprisingly, a distinctive notch remained at 7.5° (Figure 5Ci). The notch in the acceptance angle function disappears when the radial RI gradient profile is normalized to the changing cone diameter (Figure 5Ci, green line), as seen in the biological system.

Examining the fraction of rays exiting the cone after undergoing TIR (Figure S4, Supporting Information) shows that the proportion of TIR rays increases at higher angles of incidence for all models (Figure 5Cii). To test the influence of TIR on the acceptance angle, we explored the effect of increasing the RI values of the medium covering the exposed cone (which would be representative of pigment granules in the distal pigment cells) and the intercone. Increasing the RI around the exposed cone tip substantially narrowed the acceptance function of the graded RI models (Figure S5A, Supporting Information) and could provide a closer match to the experimentally measured acceptance function for the biological relevant gradient (radial model) (Figure 5Di). Although changing the intercone RI had little effect on either model (Figure S5B, Supporting Information), when the inner medium was set to 1.34, changing the intercone RI at high medium RI (*n* = 1.50), allowed us to find conditions (intercone RI of 1.42 to 1.44, Figure 4Dii) that closely match the physiologically measured acceptance function.

## 3 Conclusion

Here, we have addressed the structure–property–function relationships in the *L. polyphemus* cornea by correlating spatially resolved quantitative RI measurements with structural and compositional analysis on multiple length scales. We show that the observed RI gradients have both a structural as well as a compositional origin. Most importantly, our results point toward the contribution of ordered proteins which decorate the fibrillar chitin scaffold leading to enhanced birefringence (Δ*n* = 0.01). These proteins are co‐ordered with the chitin fibers and thus adopt the helicoidal arrangement. The nested arrangement of the helicoidal layers with increasing lamella inclination from the center to the edge of the cone, in turn leads to the observed radial structure‐based RI gradient. The RI gradient is enhanced by compositional variations that include Br doping, gradients in chitin to protein volume fraction, and in hydration level, the latter governed by sclerotization. Sclerotization and associated cross‐linking is well known for its role in stiffening the cuticle, here however, this toolkit is opted to tune the material's optical properties. The radial RI gradient is needed for on‐axis light focusing into the rhabdom. Water also plays a key role in determining the RI in the intercone region, where bulk aqueous solution is present in the multiple pore‐canals that span this region leading to the RI contrast that enables TIR of incoming light at higher incident angles while ensuring structural integrity. Interestingly, although the cone shape seems optimal for TIR, as discussed by Levi‐Setti et al., thereby increasing the acceptance angle, this effect is to a large extent suppressed by the presence of screening pigment.^[^ ^9^ ^]^ The optical properties and behavior of the cornea are therefore guided by different mechanisms at the different hierarchical length scales. The *L. polyphemus* cornea is indeed a fascinating example of the flexibility and multifunctionality of arthropod cuticle, based on a simple “blue‐print” and made from modular building blocks.

## 4 Experimental Section

### Sample Preparation

*Limulus polyphemus* specimen were obtained from the Marine Biological Laboratory (Woods Hole, MA, USA), anesthetized and sacrificed and stored at −20 °C until use. Ethical approval is not required for the use of *L. polyphemus* according to the German federal law 2013,^[^ ^40^ ^]^ however, care has been taken to prevent animal suffering. The specimens were then partially thawed at 8 °C and the lateral eyes were excised. Cells and pigments were carefully removed using fine tweezers. The remaining soft material was washed off using water spray from a syringe and a soft brush. The cleaned cornea specimens were sterilized in 70% EtOH for 24 h and transferred to ddH_2_O.

Large pieces of the cornea were placed in a silicone mold and covered in O.C.T medium (VWR Chemicals, Radnor, PA, USA). The samples were frozen within the cryo‐microtome (560 CryoStar Cryostat, Thermo Fisher Scientific, Waltham, MA, USA) and 5 to 50 µm thick section were cut using Surgipath DB80 LX blades (Leica, Wetzlar, Germany) (sample −15 °C, blade −11 °C). The sections were transferred to a glass slide using a brush, thawed, and rinsed 3× with ddH_2_O to remove excess O.C.T. Section samples were used for Raman microspectroscopy, confocal laser scanning microscopy (CLSM), QPI, FTIR mapping, and XRD/XRF.

Thin sections for Mallory staining were prepared at the CMCB‐EM and histology facility. Cornea pieces were embedded in epon, sliced 1 to 2 µm thick using an ultramicrotome and mounted on glass slides.

### Confocal Laser Scanning Microscopy

To investigate the chitin fiber architecture, 5 µm sections of cornea dyed with 0.4 mg mL^−1^ DY96 (Sigma–Aldrich, St. Louis, MO, USA) in water placed inside a µ‐dish (ibidi GmbH, Gräfelfing, Germany) were used. DY96 only emits a detectable fluorescence signal when the chitin fibers are parallel with the imaging plane.^[^ ^18^ ^]^ The dye is also known as diphenyl Brilliant Flavine 7GFF and is typically applied to polysaccharides including cellulose or chitin.^[^ ^41^ ^]^ The specificity was tested by recording the emission spectra of stained cuticle and chitin.

Imaging data was acquired with a SP‐8 laser scanning microscope (Leica) equipped with a 63× water objective (NA = 1.2). A multiphoton laser (Spectra‐Physics, Stahnsdorf, Germany) at 780 nm was used for excitation and the signal was recorded on a HyD detector between 540 and 580 nm.

### Micro Computer Tomography

µCT was performed on hydrated cornea portions stained with iodine vapor for 12 h. The stained samples were placed in a plastic vial and kept in contact with water to allow full hydration of the sample during the measurement. Transmission scans were acquired using an EasyTom160/150 equipped with a microfocus X‐ray source using a tungsten filament and a digital flat panel detector (RX Solutions, Chavanod, France). The acceleration voltage was set to 70 kV, with a current of 100 µA recording 1120 images at full rotation with a total measurement time of 3.7 h at a sample‐to‐detector distance of 773.193 mm yielding a voxel size of (2.18 µm)^3^. Reconstruction of the final volume was performed using the X‐Act software (RX Solutions, Chavanod, France).

### Fourier Transform Infrared Spectroscopy

FTIR data was acquired using a Lumos II micro‐spectrometer equipped with a nitrogen‐cooled mercury cadmium telluride detector in transmission (Bruker, Billerica, MA, USA). To reduce noise from water vapor and CO_2_, a chamber with continuous N_2_ was placed around the microscope stage. Thin cornea sections of 5 µm were placed on CaF_2_ windows (Korth Kristalle GmbH, Altenholz, Germany) for measurements. For background correction, spectra of the sample carrier were acquired by averaging 32 interferograms with a step size of 4 cm^−1^ each time before measuring the sample. Subsequently the transmittance spectrum of the sample was recorded in the same manner. For measurement control and data analysis Opus spectroscopy v8.5.29 software was used. The transmission spectra were converted into absorbance spectra and the ratio of the peaks between 1700 and 1600 cm^−1^ and 1180 and 1000 cm^−1^ (Figure S3C, Supporting Information).

### Micro‐Raman Spectroscopy

Micro‐Raman spectroscopy was performed on 10 µm hydrated cryosections mounted between a glass slide and cover slip and sealed with nail polish. An alpha300 confocal Raman microscope (WITec, Karlsruhe, Germany) equipped with a P‐500 piezo‐scanner (Physik Instrumente, Karlsruhe, Germany) was used. The laser (*λ* = 532 nm) was focused by a 40× objective lens (NA = 1.2, Nikon, Tokyo, Japan) with a laser power of 15 mW on the sample. Spectra were acquired by a thermoelectrically cooled CCD detector (DU401A‐BV, Andor, Belfast, North Ireland) behind a 600 g mm^−1^ grating spectrograph (UHTS 300, WITec, Ulm, Germany) with a spectral resolution of 3 cm^−1^. The WITec Control software (Version 5.2, WITec, Ulm, Germany) was used for measurement setup and control. The chitin, protein, and hydration maps were generated using the WITec Project Five v5.2 software by mapping the integrated intensity of the beta‐sheet peaks between 1576 and 1635 cm^−1^, the chitin amide I between 1640 and 1750 cm^−1^ and the CH band of aromatic and aliphatic side chains between 3035 and 3095 cm^−1^, and the OH bands between 3.111 and 3.689 cm^−1^.^[^ ^20^, ^21^, ^23^ ^]^

### Mallory Trichrome Staining

For Mallory staining microtome sections of 1 and 2 µm thickness were created from epon‐embedded cornea pieces and transferred (using 10% acetone) to glass slides which were dried over night at 40 °C. Sections were incubated 30 min in NaOH/100% EtOH to etch the epon followed by 3× washing in 96% EtOH and 10 min under running ddH_2_O. This was followed by 15 min at 60 °C in etchant (1:1 potassium dichromat in ddH_2_O and 10% HCl in 96% EtOH) and 10 min under running tap water. After 1 min in ddH_2_O the Mallory trichrome staining (1 g phosphotungstic acid, 2 g Orange G, 1 g aniline blue, 3 g fuchsin acid in 200 mL ddH_2_O) was applied for 15 min at 60 °C. After brief washing in ddH_2_O the glass slides containing the sections were subsequently briefly bathed in 70% EtOH and 3× in 96% EtOH. Finally, the sections were immersed in 100% EtOH for a maximum of 1 min, followed by two 2 min immersions in xylol. The samples were then dried and sealed with Cytoseal.

### Refractive Index Mapping

RI mapping was performed using a Q‐Phase microscope (TELIGHT, Brno, Czech Republic), employing QPI based on holographic microscope with low‐coherent illumination.^[^ ^28^ ^]^ During a measurement, the phase shift caused by the sample is retrieved, and used to calculate the sample RI using the relation:

(S1) $$\begin{array}{c} n_{s}=\frac{\varphi \lambda }{2\pi h_{s}}+n_{m} \end{array}$$

where *n*_s_ is the sample effective RI, *h*_s_ the local sample thickness, *φ* the measured phase shift, *λ* the light wavelength (here 650 nm), and *n*_m_ RI of the surrounding medium. In order to overcome uncertainties related to sample thickness, the measurements were carried out using two media with a different RI. The RI of the sample can then be calculated using the following equation derived from Equation (S1):

(S2) $$\begin{array}{c} n_{s}=\frac{\varphi_{2}n_{m1}-\varphi_{1}n_{m2}}{\varphi_{2}-\varphi_{1}} \end{array}$$

where indices 1 and 2 refer to the two different media used.^[^ ^29^ ^]^

Sections of 5 µm thickness were glued to a glass slide using a small drop of UHU max repair (UHU, Baden‐Baden, Germany) to prevent movement during medium exchange and covered by a sticky‐Slide (ibidi GmbH) with a 200 µm high channel. To change the medium RI, Optiprep (60% Iodixanol in water, *n* = 1.429 at 591 nm, Stemcell Technologies, Köln, Germany) at different dilutions was used.^[^ ^42^ ^]^ The RI (at 650 nm) of the iodixanol solutions was determined using an Abbe refractometer with a white light source and a 650 nm filter. During QPI measurements, medium exchange was performed using a syringe connected by Luer connectors and silicone tubing to the perfusion slide. The sample chamber and connections were sealed using parafilm. After obtaining the phase map at different ROI, the channel was flushed with 3 mL of the new solution to ensure complete exchange of medium before continuation of measurements. Data analysis was performed using in‐house python scripts and CV2 libraries for alignment of the images and calculation of RI maps.

In order to define the cornea RI gradients, datasets extracted from profile plots from cornea cross‐sections obtained at various heights along the length of cones from subsequent sections of a single animal with Fiji were seleceted.^[^ ^43^ ^]^ The profiles were ordered from distal to proximal depending on section number and cone diameter. The comparison of maximum RI of longitudinal and cross cornea sections (Figure S3A, Supporting Information) was performed on sections from cones in close proximity from a single animal mounted on the same slide. Pairs of maximum RI values were created based on the height along the cone of the respective cross‐section and the height of the profile taken on the longitudinal section.

RI of intercone: The phase‐decoupling method provides a quantitative measure of the solid material but effectively disregards free medium. The measured values for the intercone region containing water‐filled pore‐canals were corrected based on the pore canal density estimated by thresholding CLSM images of cornea cross‐sections (Figure S1C, Supporting Information). Pore canal volume fraction was estimated by measuring the ratio of masked pixels to total area. This resulted in pore canal density around 15%. Assuming RI of 1.34 for the pore canal filling medium, the final RI of the intercone regions obtained was 1.44.

### X‐Ray Diffraction and X‐Ray Fluorescence

Cone sections of 50 µm thickness, prepared using a cryo‐microtome (see above), mounted on SiN windows, were measured at mySPOT beamline at BESSY II, HZB (Helmholtz‐Zentrum Berlin für Materialien und Energie, Berlin, Germany) and at ID13 beamline at ESRF (European Synchrotron Radiation Facility, Grenoble, France). In both cases the beam energy was set to 15 keV (0.827 Å), a diffraction detector in transmission geometry with a sample‐to‐detector distance of around 300 mm, and fluorescence detector at roughly 90° relative to the beam was used.

At the mySpot beamline the beam energy was set with a B4C/Mo Multilayer monochromator and an Eiger 9 M (pixel size (75 × 75) µm^2^, Dectris, Baden, Switzerland) XRD detector was used with a (30 µm)^2^ beam. Quartz was used for detector calibration and a single element Si(Li) XRF detector (RAYSPEC (SGX Sensortech) was used for XRF. For hydrated maps, sections in water were placed in‐between SiN windows sealed with vacuum grease to prevent evaporation. Oversampling with step size of 8 µm was used in both directions at an acquisition time of 10 s.

At the ID13 beamline the beam energy was set with a Si(111) monochromator and an Eiger 4 M (pixel size (75 µm)^2^, Dectris) XRD detector was used with a focal size of (300 nm)^2^. Quartz and silver behenate powders were used for detector calibration. A Vortex EM, silicon drift detector with 50 mm^2^ sensitive area was used for recording the XRF signal. Maps were acquired with a step size of 1 µm in *x* and *y* and an acquisition time of 100 ms.

XRF maps were generated from integrating the BrK*α* peak intensity and then corrected by primary beam intensity. XRD data reduction was performed using DPDAK v1.4.1 to obtain XRD 1D profiles.^[^ ^44^ ^]^ Background and primary beam correction were performed using in‐house python scripts based on the numpy library. Fiber correlation peak fitting was performed using dpdak. Protein/chitin volume fractions and water content were calculated using Equation (1).^[^ ^22^ ^]^ The profiles shown in Figure 2 were averaged over ≈100 diffraction patterns in the corresponding region using in‐house python scripts using sympy library.

Water volume fraction: The calculation of the water volume fraction follows the assumption that bound water is retained in the air‐dried section, and the total volume is a sum of chitin and proteins. In the hydrated samples the total volume consists of chitin, proteins, and water. It was assumed that the ratio of chitin and proteins is unchanged, and the hydrated protein fraction was calculated from the hydrated chitin fraction multiplied by this ratio (Equation 2).

Chitin crystallite size: The in‐plane fiber orientation can be retrieved from the orientation of the correlation peak on the 2D detector, readily confirming the nested organization of the helicoidal lamellae seen with CLSM (Figure S2A, Supporting Information). To confirm that chitin crystallite size is uniform across the cornea, averaged XRD spectra from different regions across the cone were fitted. For this, the (110) peak was fitted with four Gaussians to account for the additional signal from other chitin and protein reflections (Figure S2B, Supporting Information). The fitting parameters of the main peak were used to solve the Scherrer equation

(S3) $$\tau =\frac{K\times \lambda }{\beta \times \cos\left(\theta \right)}$$

where *τ* is the crystallite thickness, *K* is a form factor (here set to 1), *λ* the wavelength used (0.82656 Å), *β* the FWHM, and *θ* the scattering angle of the reflection. The average result for chitin crystallite thickness from 14 different regions within the cornea was 24.52 ± 2.37 Å. Assuming a distance of *b* = 18.9 Å for the respective crystallographic plane, this leads to a chitin fiber diameter of 28.35 Å or 1.5 unit cells in this direction.^[^ ^19^ ^]^ This slight offset is most likely due to the large number of degrees of freedom resulting from the simultaneous fitting of four peaks.

### Model Generation for FDTD and Ray‐Tracing Analyses

The µCT volume data was segmented using Amira (v5.3) to label the extent of six cones, as well as the surrounding intercone, outer‐cornea, and epicornea. A Matlab script was developed to calculate the radially averaged surface profile along each cone, as well as its outer‐cornea protrusion, epicornea dent, and the bordering of internally exposed intercone. The angle between the central axis of the cone and corneal normal differed by 22.2° ± 1.5° in the section of the cornea that was scanned. To remove the influence of off‐axis optics from the analysis, the surfaces of the outer‐cornea and epicornea to each cone were aligned by rotating the corneal normal to align with its central axis and then translated the tip of the cone‐in‐cone and epicornea cone so that they were on the axis. The internally exposed intercone surface lies in parallel to the cornea and was also rotated to align it with the central axis of each cone, however, as the intercone surface also follows the cone shape, a shear transformation then had to be applied to the intercone so that its alignment remained consistent with the cone surface after rotation. Finally, the outer‐cornea protrusion and epicuticle dent were shifted such that the cone axis passed through their center.

As the length of each of the six analyzed cones varied slightly (215.4 ± 7.4 µm), the radial outlines of each structure were stretched to a uniform length before calculating an average radial outline, which was stretched back to the average cone length (Figure S5C, Supporting Information), and finally smoothed. The averaged radial profile of each structure was used to generate either 2D RI maps at (500 nm)^2^ resolution for FDTD simulations or 3D RI maps at (2 µm)^3^ resolution for ray‐tracing simulations. Triangulated meshes were also calculated to delineate the border of each surface. Unless otherwise noted, the RI map used the following values for each structure: outer media – 1.33; epicornea – 1.53; outer‐cornea – 1.5; intercone – 1.40; cone – 1.52; inner media – 1.34. In addition to using a constant RI value for the cone, the following RI profiles were also modeled:

Observed: *RI* (*r*, *z*) = 1.5 + (*z* × 0.01 + 0.01) − (0.5 × *z* + 0.8) × 0.0392 × *r* ^2^, where *z* is the vertical distance from the cone tip (normalized to 1) and *r* is the radial distance from cone center line (normalized to 1, relative the radius at each step along the profile).

The linear component of the observed profile could be modeled separately by setting *r* = 0. Likewise, the radial component could be modeled separately by setting *z* = 0 or 1 for the cone tip or base, respectively (Figure S4A, Supporting Information).

Theoretical:, i.e., the parabolic RI profile required to focus light at the tip of a lens cylinder of arbitrary length.^[^ ^8^ ^]^ The profile was defined as: $RI\;\left(r\right)=1.52\times \mathrm{sech}\left(\frac{\pi \;r}{l}\right)$, where *r* is the absolute radial distance from the cone center line and *l* is the absolute length of the cone. Besides modeling a cone with this theoretical RI profile, a full cylinder, with a radius equal to the maximum of the average radial cone profile was also modeled.

### Ray Tracing

3D ray tracing was performed by implementing the simulation procedure for media with discretely defined isotropic but inhomogeneous RI developed by Nishidate et al. in a Matlab script.^[^ ^45^ ^]^ The following two modifications were made to the procedure published by Nishidate et al.: 1) Numerical integration was performed using the 4^th^ order Runge–Kutta scheme described by Sharma et al. with a fixed step size of 0.5 µm, rather than the Runge–Kutta–Fehlberg scheme with an adaptive step size;^[^ ^46^ ^]^ 2) refraction at lens surfaces was calculated based on the ray's intersection to the triangulated mesh of the lens surface, rather than using a Nagata patch. Our implementation achieved comparable accuracy to that reported by Nishidate et al. when tested on a Luneburg lens and an optical fiber with a parabolically graded RI.^[^ ^45^ ^]^ Simulations were conducted for rays placed at 10 µm intervals on two‐dimensional grid and for angles (relative to the cone axis) from 0° to 20° spaced by 2.5° intervals. Rays that did not enter the base of the cone (i.e., rays that did not enter the cone or entered along its side) were not included in the analysis, nor were rays that reversed direction due to TIR.

Ray‐tracing results were described by plotting orthogonal perspectives of ray paths (paths are shown for rays at 20 µm intervals [i.e., every 2^nd^ ray simulated] to preserve readability) and spot diagrams of the position of all rays on a plane passing through the cone tip. Anatomical studies indicate that the cone is sheathed in pigment until approximately 15 µm away from the cone tip, while the top of the receptor is positioned 5 µm proximal to the cone tip and has a radius of 25 µm in dark‐adapted animals at night (see the top diagram in Figure S4B, Supporting Information).^[^ ^47^ ^]^ As such, the height and radius of the best focus were calculated in both perspectives as well as for the circle of least confusion (COLC), from rays that exited the cone less than 15 µm from its tip. The approximate receptor acceptance function was calculated as the number of rays that exited the cone tip and then entered the receptor within its dark‐adapted radius for each angle of incidence (which was normalized to a fraction based on the number of rays entering the receptor at 0°). This approximation represents an upper bound on an acceptance function, as the proportion of light absorbed from each ray by the receptor will vary depending on the rays entry position and angle. A plot of relative sensitivity as a function of illumination angle (for night adapted receptors) measured from optic nerve fibers by Barlow et al. (Figure 4C) was digitized using WebPlotDigitizer and averaged around 0°; this provides an independent reference for our acceptance function calculations.^[^ ^39^ ^]^ An acceptance function based on the day‐adapted receptor dimensions and pigment aperture (see the bottom diagram in Figure S4B, Supporting Information) was also calculated, and found that light only reached the receptor at normal incidence. This is a limitation caused by the resolution used in the ray‐tracing model, as the half‐width measured for the physiological light‐adapted acceptance function is 3^o^.^[^ ^39^ ^]^

### Finite‐Difference Time‐Domain (FDTD) Simulations

Light scattering by the internal structure of the cones was simulated using a two‐dimensional FDTD method in Lumerical FDTD 2012 R1.4 (Ansys Canada Ltd., Vancouver, Canada; https://www.lumerical.com/products/fdtd/), a commercial‐grade FDTD simulator. FDTD simulations were performed based on the different models obtained from µCT scans as explained above. The incident light beam was assumed to be a plane‐wave with a broad‐band spectrum centered around 650 nm, the wavelength at which the RI maps were obtained. The electric field maps were calculated from light flux through the structure.

### Statistical Analysis

For XRD/XRF, FTIR, microCT, CLSM, and Mallory staining 3 to 10 specimens from six animals were investigated. For micro‐Raman and hydrated XRD samples, several sections from a single specimen were used. Image and data processing and analysis was performed as described in Experimental Section. Where indicated, the values were presented as means and standard deviations (mean ± SD). The statistical analysis and data processing were carried out using available python libraries.

## Conflict of Interest

TS and MAKAR are employed by TELIGHT. All other authors declare that they have no competing interests.

## Authors Contribution

Y.P., G.S., O.S., and P.F. worked on conceptualization. Y.P., P.F., and W.W. performed project administration. O.S., E.S., M.S., J.L., and Y.P. performed XRD/XRF experiments, O.S., MAKAR, T.S. performed QPI experiments. C.S. and O.S. performed Raman experiments. O.S., G.J.T., L.B., and Y.P. performed data analysis. B.D.W. performed FDTD modeling and simulations. G.J.T. performed ray tracing. Y.P., O.S., G.J.T. and B.D.W. wrote the manuscript with contribution from all other authors. Open Access funding enabled and organized by Projekt DEAL. [Correction added on November 24, 2022, after first online publication: Projekt Deal funding statement has been added.]

## Supporting information

Supporting information

## Data Availability

The data that support the findings of this study are available from the corresponding author upon reasonable request.
