# Supplementary material for: Gradients of Orientation, Composition, and Hydration of Proteins for Efficient Light Collection by the Cornea of the Horseshoe Crab
Source: Adv Sci (Weinh). 2022 Oct 17;9(33):2203371. doi: 10.1002/advs.202203371 (PMC9685478; doi:10.1002/advs.202203371)
Supplement: Supplementary file 1 — Supporting information [file ADVS-9-2203371-s001.pdf]

ADVANCED SCIENCE
Open Access

Supporting Information

for *Adv. Sci.*, DOI 10.1002/advs.202203371

Gradients of Orientation, Composition, and Hydration of Proteins for Efficient Light Collection by the Cornea of the Horseshoe Crab

*Oliver Spaeker, Gavin J. Taylor, Bodo D. Wilts, Tomáš Slabý, Mohamed Ashraf Khalil Abdel-Rahman, Ernesto Scoppola, Clemens N. Z. Schmitt, Michael Sztucki, Jiliang Liu, Luca Bertinetti, Wolfgang Wagermaier, Gerhard Scholtz, Peter Fratzl and Yael Politi**

Supporting Information

Gradients of Orientation, Composition and Hydration of Proteins for Efficient Light Collection by the Cornea of the Horseshoe Crab

*Oliver Spaeker, Gavin J. Taylor, Bodo Wilts, Tomáš Slabý, Mohamed Ashraf Khalil Abdel-Rahman, Ernesto Scoppola, Clemens NZ Schmitt, Michael Sztucki, Jiliang Liu, Luca Bertinetti, Wolfgang Wagermaier, Gerhard Scholtz, Peter Fratzl, and Yael Politi**

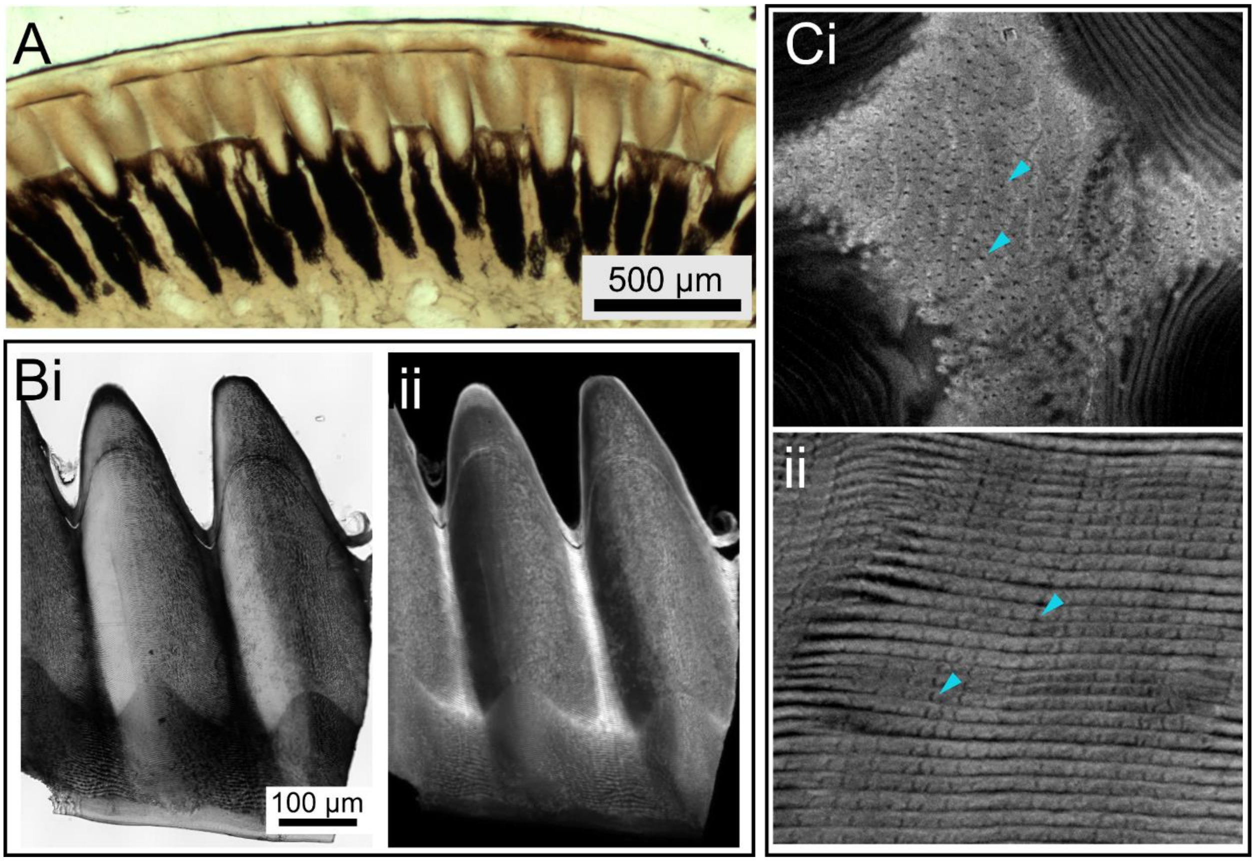

Figure S1. Cornea sections: lamellae and pore canals. (A) Longitudinal section through untreated cornea embedded in PMMA shows the corneal cone arrangement and the pigment enveloping the cones. The amber color results from the embedding procedure. The cornea is typically light amber and transparent. **(B)** Low magnification of cornea longitudinal section imaged using CLSM (i) bright-field image and (ii) DY96 channel. Note that the epicornea is unstained indicating the absence of chitin. **(C)** Magnified intercone regions showing multiple pore canals (dark structures, two are indicated by arrows) in (i) cross section and (ii) longitudinal sections. The lamellated organization of the material in the cones (Ci) and the intercone region (Cii) is also clearly visible.

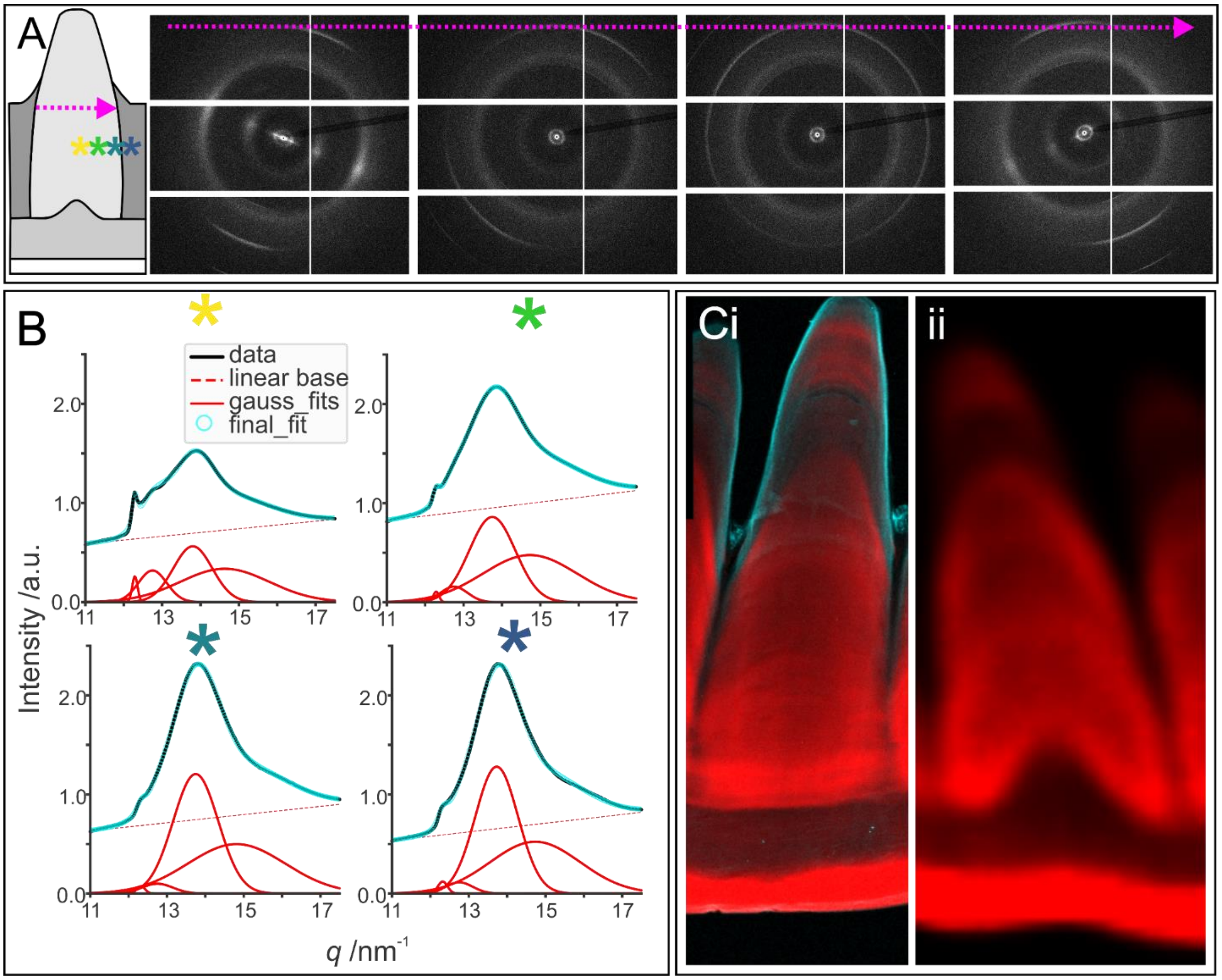

Figure S2. XRD/XRF analysis of cone longitudinal sections (A) 2D diffraction patterns across a longitudinal cornea section showing the typical chitin pattern and the fibrous nature of the cuticular cornea. The rotation of the anisotropic fiber signal confirms the nested helicoidal structure observed by CLSM: the pink arrow indicates the direction across the cone from which the diffraction patterns were taken. Colored asterisks indicate the positions of plots in B. **(B)** Fitting on the (110) reflection to obtain parameters for calculating the chitin crystallite thickness in the cornea by the Scherrer equation. **(C)** XRF maps of Br (red) and Zn (cyan) (i) shows the Zn layer surrounding the exposed part of the cone. The section does not contain the outer-cornea protrusion due to oblique sectioning. (ii) Low resolution map of a longitudinal

section through the center of the cone, showing the distribution of Br around the outer-cornea protrusion.

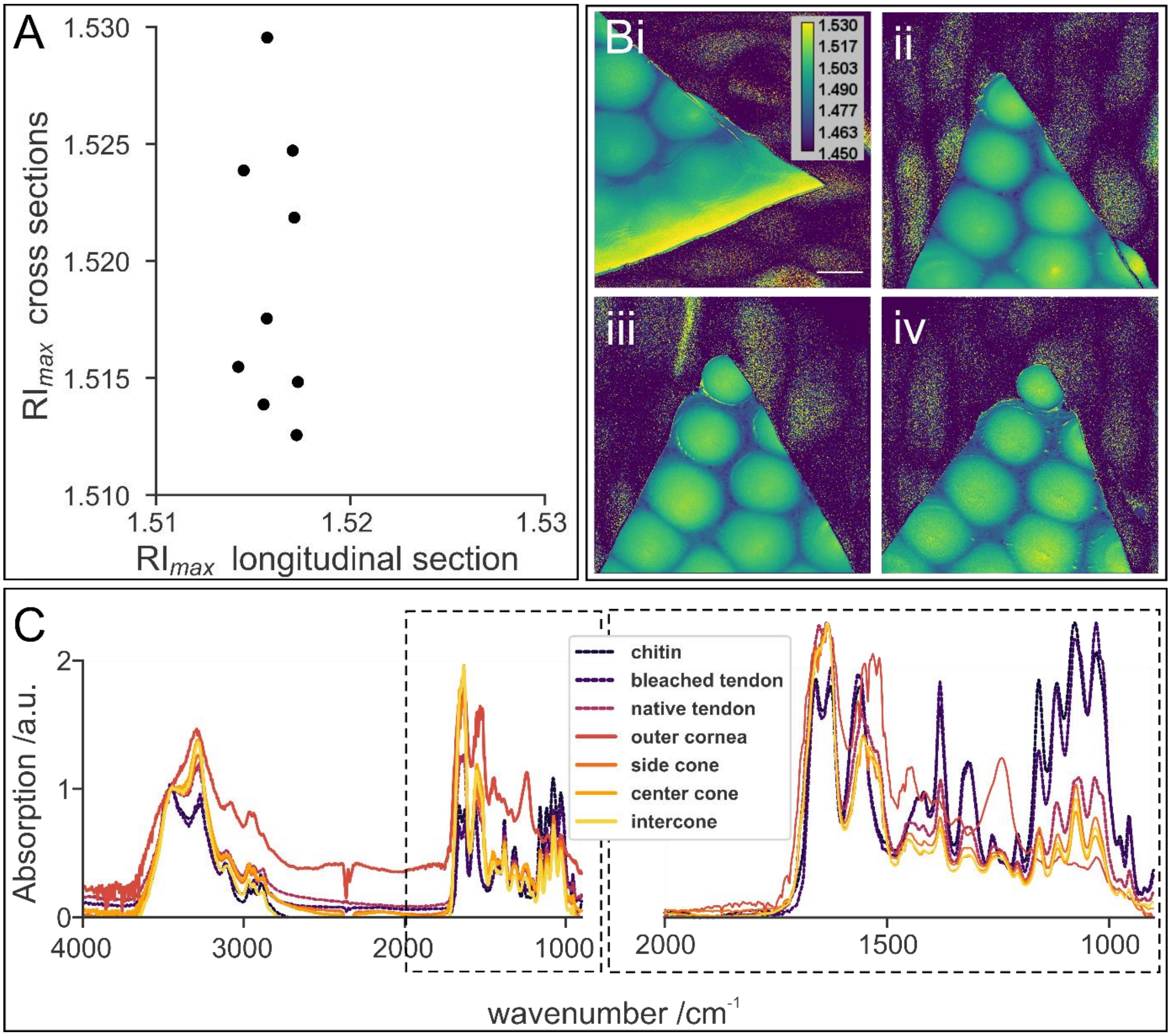

Figure S3. RI mapping and compositional variation (A) Pairs of maximum RI values of cross and longitudinal sections of the same animal, sectioned from corneal cones in close proximity and measured at the same experimental session. Pairs were chosen to be at approximately the same height along the cone. **(B)** RI maps of a cornea cross section in a curved cornea region, allowing access to the outer cornea (top left). **(C)** FTIR spectra of chitin reference as well as

native and deproteinized *L. polyphemus* tendons compared with FTIR spectra of different regions of the cornea. The full range is normalized to the peak at 3450 cm^{-1}, the spectra of the magnified plot are normalized to their respective maximum values, emphasizing the individual contribution of chitin and protein. The map in Figure 2G shows the ratio of the integrated signal of chitin and protein over the integrated chitin intensity (1700 cm^{-1} to 1600 cm^{-1} over 1180 cm^{-1} to 1000 cm^{-1}).

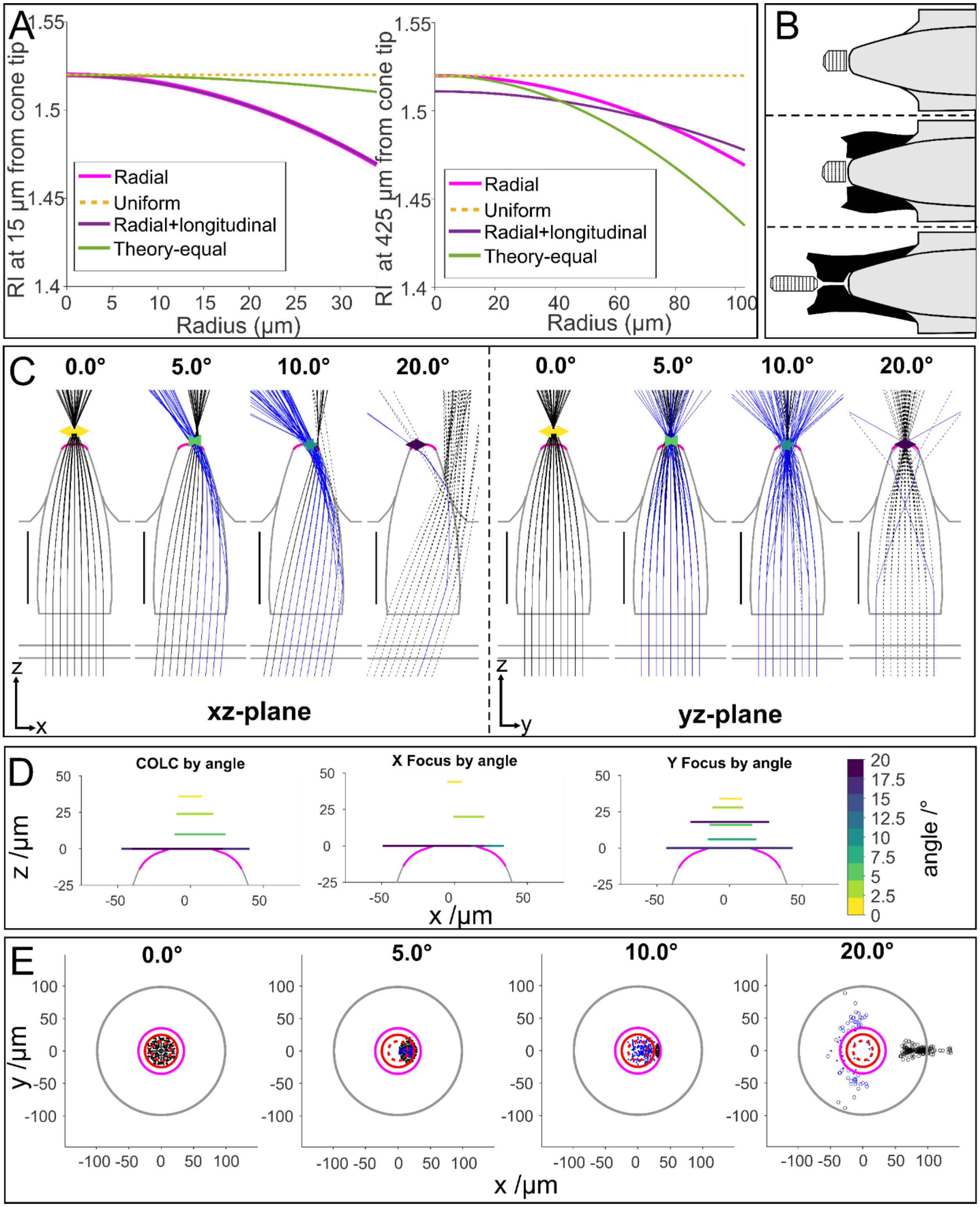

Figure S4. 3D Ray-tracing methodology. (A) RI profiles used for ray tracing. The left plot is calculated at the tip of the exposed cone, the right plot is at the widest (base) part of the cone.

(B) Receptor position and pigment (depicted in black) defining the aperture in light or dark adaptation according to Chamberlain and Barlow 1987 top to bottom: dark adapted model with low inner medium RI (*n*=1.34), dark adapted with pigment (n=1.50 for inner medium), light adapted with narrow elongated aperture. **(C)** Ray focusing in the xz- and yz-plane on the example of 'radial' gradient model for different incident angles (the same model as shown in Figure 4B).[47] The pink outline on the cone tip shows the exposed cone tip (also shown on the outlines in D and E). The circle of least confusion (COLC) is depicted as a colored horizontal arrowed bar, rays that undergo TIR are plotted in blue (also shown in E), and rays that exit the cone before its tip are dashed (open circles in E). **(D)** Same model as in C showing lines denoting the position and diameter of the COLC, X focus and Y focus for all angles simulated (note that the focal heights usually descend to the cone tip at angles greater than 7.5°, in which case the lines overlap). The color bars denote the incidence angle in degrees. **(E)** Spot diagrams (at the cone tip) for the same cone model, showing the rays (dots) exiting the cone tip (pink circle) and entering the receptor (night – solid red circle, day – red dashed circle) for selected angles. The cone base is represented by the grey circle.

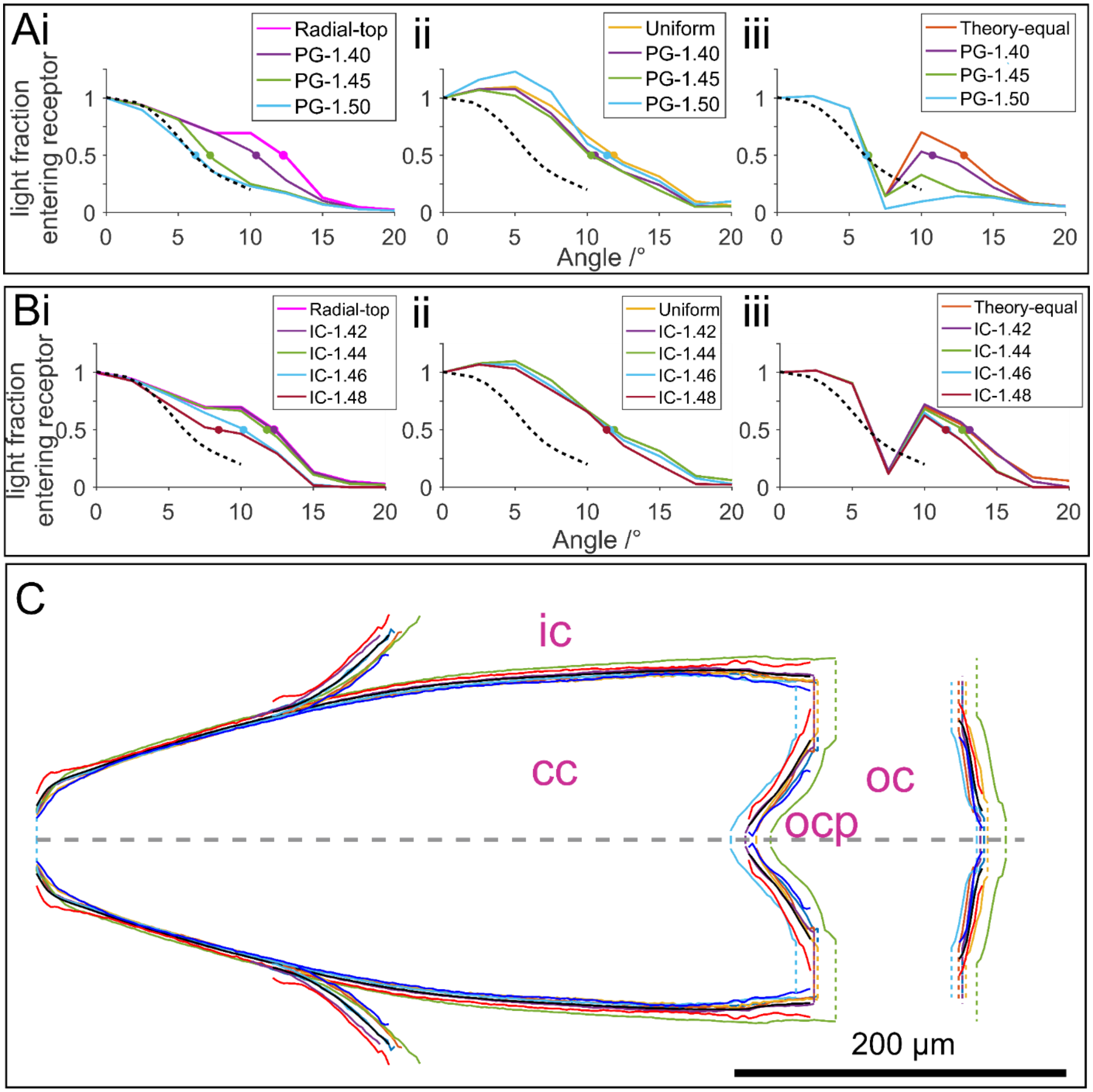

Figure S5. 3D Ray-tracing results for variation of pigment and intercone RI. (A) Effect of varying RI of screening pigment (PG) around exposed part of cone on acceptance function for three different models (intercone RI: *n*=1.40). **(B)** Effect of varying the RI of the intercone (IC) on acceptance function for three different models (inner medium RI: *n*=1.34). **(C)** Diagram showing variation in the cone outline measured from six segmented groups of the corneal cone (cc), outer-cornea protrusion (ocp), intercone (ic) and outer cornea (oc). Black lines indicate the averaged profile, while the blue and red lines indicate the mean minus or plus 4 SD, respectively.

The dashed grey line represents the central axis (and average length) of the cone around which all profiles are symmetric.
